# Potential lesson from a model-based exploration on treatment effect heterogeneity of mal de débarquement syndrome

**DOI:** 10.3389/fneur.2025.1648253

**Published:** 2025-10-06

**Authors:** Jun Maruta, Sergei B. Yakushin, Catherine Cho

**Affiliations:** ^1^Department of Neurology, Icahn School of Medicine at Mount Sinai, New York, NY, United States; ^2^Department of Rehabilitation and Human Performance, Icahn School of Medicine at Mount Sinai, New York, NY, United States; ^3^Department of Neurology, NYU Grossman School of Medicine, New York, NY, United States; ^4^Department of Otolaryngology, NYU Grossman School of Medicine, New York, NY, United States

**Keywords:** central vestibular disorder, dizziness, gravity, imbalance, optokinetic, orientation vector, vestibular habituation, vestibulo-ocular reflex

## Abstract

**Background:**

A central vestibular neural mechanism known as velocity storage may be inappropriately conditioned in mal de débarquement syndrome (MdDS), a rare chronic vestibular disorder with a continuous false sensation of self-motion described as non-spinning vertigo. Visual-vestibular therapy approaches designed to recondition the three-dimensional properties of velocity storage have yielded much clinical success, but not without limitations. An alternative therapeutic approach, designed to attenuate the contribution of malfunctioning velocity storage in higher-order neural processing, has also yielded positive results, but at a lower success rate. We sought a possible explanation for the latter shortcoming using a mathematical model.

**Methods:**

The three-dimensional orientation properties of velocity storage can be modeled as a dynamical system using a 3 × 3 system matrix. For normal upright, the system matrix is diagonal, with its eigenvectors aligning with the head-fixed roll, pitch, and yaw axes, and the yaw eigenvector with gravity. A pull sensation of MdDS has been expressed with a system matrix with off-diagonal elements representing cross-axis coupling and interpreted as a misalignment between the yaw eigenvector and the head vertical. We manipulated the velocity storage’s yaw time constant and output weight.

**Results:**

The model predicted that attenuating the velocity storage contribution could exaggerate the pull sensation.

**Conclusion:**

The present model-based exploration points to a possible weakness in the MdDS treatment approach focused on velocity storage attenuation, while likely beneficial otherwise. When a pulling sensation is present, the treatment protocol may need to be supplemented with another approach that specifically counters this problem, such as optokinetic stimulation.

## Introduction

Mal de débarquement syndrome (MdDS) is a rare chronic vestibular disorder with a continuous false sensation of oscillatory self-motion, such as rocking, swaying, or bobbing, or of gravitational pull as though being pulled in a particular direction, which are collectively described as non-spinning vertigo (1–4). MdDS characteristically presents with additional symptoms such as migraine, stress, depression, anxiety, and cognitive problems and is physically, psychosocially, and economically debilitating (5–7). Treatment options for the illness are limited, and medications may offer only partial or symptom-specific relief (4, 8–11).

Typically onsetting after prolonged exposure to passive motion during a voyage on a cruise ship or airplane, MdDS is a disorder that is thought to stem from neural plasticity rather than damage. Conventional vestibular physical therapy is generally ineffective in treating this illness (4, 9, 12), but the recent discovery that it may involve maladaptation of the velocity storage mechanism in the central vestibular system opened opportunities for positive long-term outcomes (13–16).

Velocity storage is an integral element of the vestibulo-ocular and optokinetic reflexes, first examined as a stored eye movement drive that prolongs the vestibular and optokinetic nystagmus beyond the input activity (17–20). Yet, nystagmus can be similarly generated and sustained without coplanar optokinetic or semicircular canal activation, such as when following a rotating wall or floor with limbs in darkness, indicating that the velocity storage mechanism reconstitutes self-motion signals from multimodal sensory inputs (21, 22). In addition to these ocular reflexes, velocity storage is also thought to contribute to postural reflexes and the perception of self-motion (17–19, 22–26).

Velocity storage’s capacity to reconstitute signals of self-motion further extends to dynamically transforming them in real time to orient to the gravito-inertial field and act as a “neural gyroscope” (27–29). For example, horizontal optokinetic nystagmus induced in a laterally tilted position gives way to optokinetic after-nystagmus (OKAN) that has a vertical component (29–31). Similarly, the per-rotatory nystagmus induced with off-center rotation in the horizontal plane while facing forward or backward in the direction of travel evolves with an out-of-plane, vertical component as the centripetal acceleration tilts the gravito-inertial field (32). Critically, for velocity storage to interpret the incoming information and perform coordinate transformations as such, it needs to maintain its own referential representation of three-dimensional space. That this referential representation is malleable and maladapted in MdDS is the central idea of the velocity storage-based postulate for the illness’s pathophysiology (13, 33–35). Unfortunately, the physical signs of MdDS are inconsistently present, and direct evidence to uphold this postulate has thus far been lacking (13, 16). Nevertheless, the success of the treatment approaches designed to readapt, or recondition, the three-dimensional spatial orientation properties of maladapted velocity storage supports the postulate that underlies these approaches (13–15, 35–38).

We recently reported that an alternative approach, designed to attenuate velocity storage’s contribution to the central vestibular pathways (rather than to readapt its spatial orientation properties), yielded a clinically significant treatment effect in about half of the patients tested (16). The rationale behind this treatment was that, if MdDS was caused by malfunctioning velocity storage, attenuating its contribution to higher-order neural processing should also reduce the symptoms of the illness. Previous experiments had shown that repeated vestibular or visual-vestibular training could attenuate velocity storage contribution to the vestibulo-ocular reflex (VOR) by shortening the duration of its activity (as measured by the decay time constant) in an effect known as habituation (39–42). Once habituated, individuals tend to retain this state over a long period of time (39, 41, 42). In our study, a modified application of a visual-vestibular habituation protocol previously deployed in motion sickness treatment (39) to patients with MdDS resulted in groupwise reductions in the velocity storage contribution as related to its output amplitude as opposed to time constant (16), notwithstanding that other habituation protocols may yield different results. Remarkably, however, most patients who responded positively to our treatment protocol continued to experience significantly reduced symptoms throughout the six-month follow-up period.

While our results were overall encouraging, it is unclear why the benefit of the treatment was not more widely applicable. In the present study, we sought possible explanations using a mathematical model. How velocity storage orients to spatial vertical by transforming the axis of eye rotation during the VOR or OKAN has been modeled as an adjustment of the orientation vector associated with the head vertical to better align with gravity (28, 29, 43). A recent analysis based on such a model aptly explained the pulling sensation that some patients with MdDS experience as a misalignment between the orientation vector and the head vertical (35). Therefore, the present analysis focused on the effect of velocity storage attenuation on the pulling sensation.

## Methods

### Normal condition

**Figure 1:**
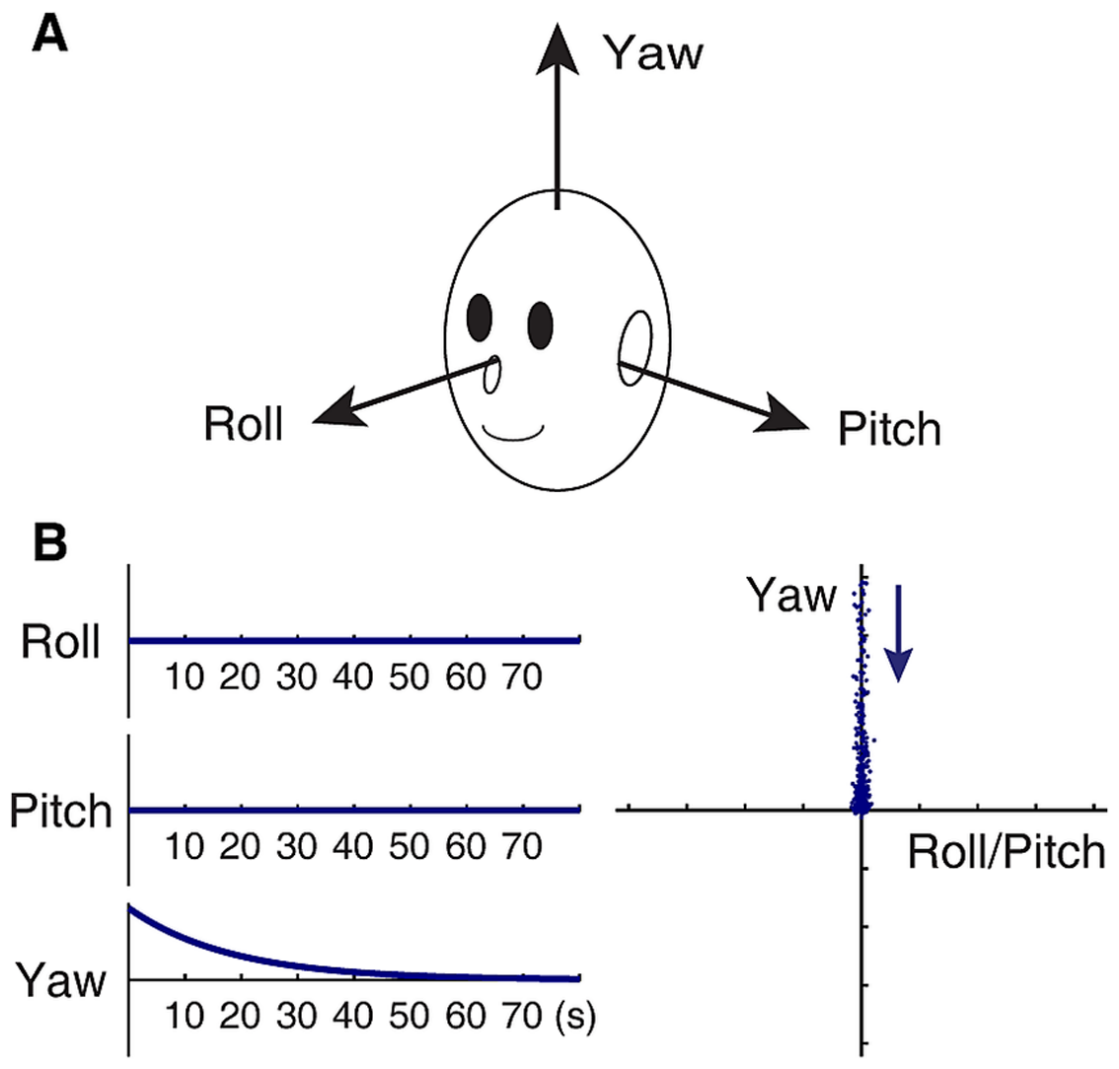
Model-based characterization of normal upright. **(A)** Head-fixed coordinate frame of reference. **(B)** Idealized slow phase velocity of OKAN subsequent to yaw OKS (zero input response) in an upright position, plotted against time in seconds (left panels), and the same plotted in the roll/pitch-yaw plane (right panel). The time constant of decay in yaw is set to 18 s. In the right panel, each dot represents slow-phase eye velocity sampled at a rate of four per second. The dots are separated by artificially injecting noise in the data to facilitate visualization of spatio-temporal progression in the direction indicated by the arrow. As a result, initial fast and later slow changes are represented by a sparse and dense display of dots, respectively.

Since its formal conceptualization, the velocity storage mechanism has been modeled as a leaky integrator (17–19, 44, 45). It was later found that velocity storage had three-dimensional orientation properties that were related to the position of the head relative to gravity, which could be modeled as a dynamical system of roll, pitch, and yaw components (28, 29, 31, 43). Thus, the dynamical system equation for the velocity storage integrator may be represented as $\dot{x}=Hx$, where x(t) is a three-dimensional vector representing the state of the system at time *t*, with its components *x_roll_*(*t*), *x_pitch_*(*t*), and *x_yaw_*(*t*) being velocity components about the roll, pitch, and yaw axes, respectively, of the head-fixed coordinate frame of reference (Figure 1A), and **H** is a 3 × 3 matrix of parameters that determine the dynamic behavior of x(t). Note that **H** has a structure that exists independently of the state of the system. The system matrix for normal upright **H**_**0**_ is given by:


$$(\begin{array}{ccc} h_{\mathrm{rr}} & 0 & 0\\ 0 & h_{\mathrm{pp}} & 0\\ 0 & 0 & h_{\mathrm{yy}} \end{array}),$$

where the velocity storage time constants for rotations about the head roll, pitch, and yaw axes are *negatively and reciprocally* related to the corresponding diagonal elements, e.g., the time constant for the yaw component is given by −1/h_yy_. The roll and pitch time constants are usually several-fold shorter than the yaw time constant and closer to those of primary afferents when gravity is aligned with the yaw axis (46–48). Eigenvectors ***u****_roll_*, ***u****_pitch_*, and ***u****_yaw_* of **H**_**0**_, respectively, represent the velocity storage estimate of the head roll, pitch, and yaw axes, aligned with the actual head roll, pitch, and yaw axes. In particular, ***u****_yaw_* defines the subjective “up” direction.

The zero-input response vector of the system (i.e., response to some initial condition without any further input), equivalent to the slow phase eye velocity profile of an idealized OKAN, is represented as:


$$x(t)=(\begin{array}{l} x_{\text{roll}}(t)\\ x_{\text{pitch}}(t)\\ x_{\mathrm{yaw}}(t) \end{array})=(\begin{array}{l} x_{\text{roll}}(0)e^{h_{\mathrm{rr}}t}\\ x_{\text{pitch}}(0)e^{h_{\mathrm{pp}}t}\\ x_{\mathrm{yaw}}(0)e^{h_{\mathrm{yy}}t} \end{array}).$$

In a normal condition, the slow phase velocity of OKAN in an upright position subsequent to yaw OKS (i.e., *x_roll_*(0) = *x_pitch_*(0) = 0) remains in the yaw axis, decaying exponentially with a time constant given by −1/h_yy_ (Figure 1B). As a matter of fact, under the stipulation that the yaw time constant is larger than the roll or pitch time constant, the tail end of OKAN generally approaches the yaw axis regardless of the OKS direction as long as *x_y_*(0) ≠ 0.

### Gravitational pull

**Figure 2:**
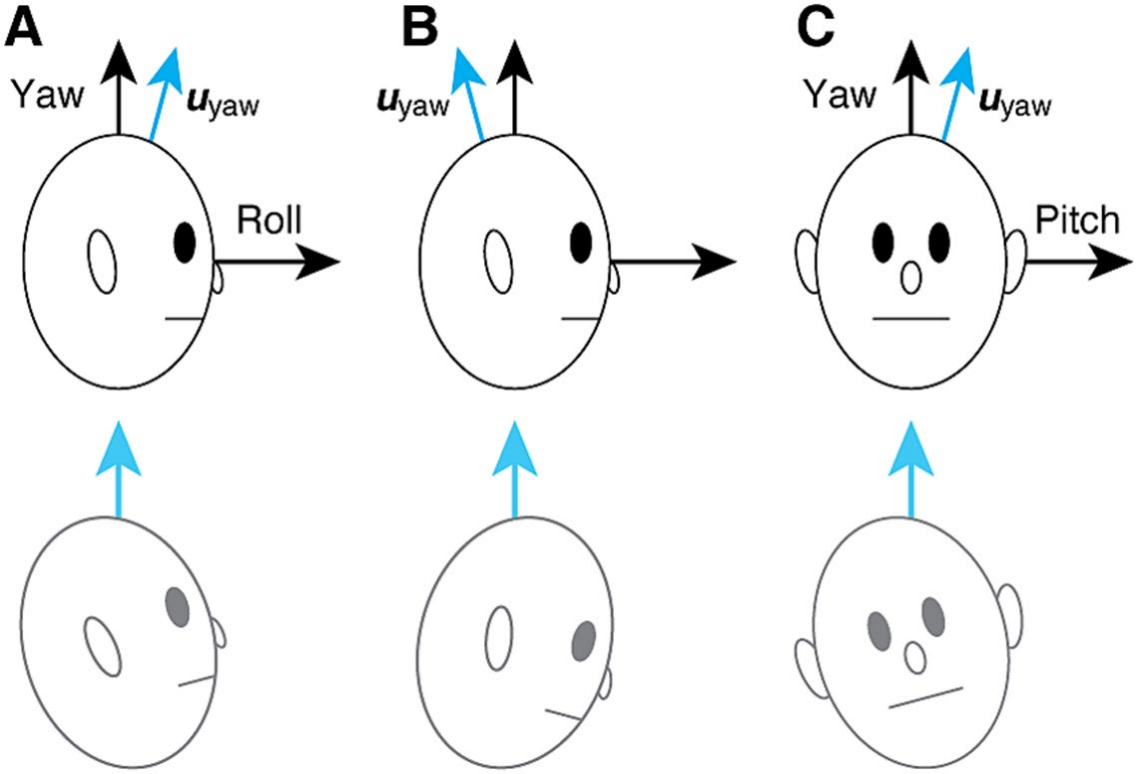
Hypothesized mechanism of gravitational pull sensation in MdDS (35). **(A)** Backward pull associated with yaw-to-roll coupling. The blue arrow indicates the subjective estimate of the up direction. The patient’s urge to align the subjective “up” with gravity is falsely experienced as a backward pull and loss of balance. **(B)** Forward pull, also associated with yaw-to-roll coupling but with the opposite polarity from **(A)**. **(C)** Rightward pull associated with yaw-to-pitch coupling. The bottom row illustrates the associated subjective experience.

The gravitational pull sensation of MdDS may be interpreted as a misalignment between the head vertical (yaw) axis and its velocity storage representation ***u****_yaw_*, resulting in an incorrect subjective estimate of the up direction (35). As such, the system matrix **H**_**pull**_ for gravitational pull can be represented as an alteration from **H**_**0**_, given by:


$$(\begin{array}{ccc} h_{\mathrm{rr}} & 0 & h_{\mathrm{yr}}\\ 0 & h_{\mathrm{pp}} & h_{\mathrm{yp}}\\ 0 & 0 & h_{\mathrm{yy}} \end{array}),$$

with the off-diagonal elements h_yr_ and h_yp_, respectively, representing yaw-to-roll and yaw-to-pitch coupling. An OKS treatment for gravitational pull, on the other hand, can be understood as reducing these elements (35). Notably, **H**_**pull**_ for gravitational pull forward or backward (Figures 2A,B) is given by:


$$(\begin{array}{ccc} h_{\mathrm{rr}} & 0 & h_{\mathrm{yr}}\\ 0 & h_{\mathrm{pp}} & 0\\ 0 & 0 & h_{\mathrm{yy}} \end{array}).$$

Similarly, **H**_**pull**_ for laterally directed gravitational pull (Figure 2C) is given by:


$$(\begin{array}{ccc} h_{\mathrm{rr}} & 0 & 0\\ 0 & h_{\mathrm{pp}} & h_{\mathrm{yp}}\\ 0 & 0 & h_{\mathrm{yy}} \end{array}).$$

In general, an eigenvector basis of **H**_**pull**_ is given by:


$$u_{\text{roll}}=(\begin{array}{c} 1\\ 0\\ 0 \end{array}),u_{\text{pitch}}=(\begin{array}{c} 0\\ 1\\ 0 \end{array}),u_{\mathrm{yaw}}=(\begin{array}{c} \frac{h_{\mathrm{yr}}}{h_{\mathrm{yy}}-h_{\mathrm{rr}}}\\ \frac{h_{\mathrm{yp}}}{h_{\mathrm{yy}}-h_{\mathrm{pp}}}\\ 1 \end{array}),$$

where ***u****_roll_* and ***u****_pitch_*, respectively, align with the head roll and pitch axes, but ***u****_yaw_*, the subjective estimate of the up direction, does not align with the head vertical. An attempt to align ***u****_yaw_* to gravity is presumed to contribute to the sensation of gravitational pull and loss of balance (Figure 2, bottom row) (35). The angle *γ* of the misalignment can be obtained from the yaw-direction cosine of ***u****_y_* given by Equation 1:


(1) $$\cos(\gamma )=\frac{1}{\sqrt{{\left(\frac{h_{\mathrm{yr}}}{h_{\mathrm{yy}}-h_{\mathrm{rr}}}\right)}^{2}+{\left(\frac{h_{\mathrm{yp}}}{h_{\mathrm{yy}}-h_{\mathrm{pp}}}\right)}^{2}+1^{2}}}$$

Finally, the zero-input response vector associated with **H**_**pull**_, equivalent to OKAN, is represented as Equation 2:


(2) $$x(t)=(\begin{array}{c} x_{\text{roll}}(0)e^{h_{\mathrm{rr}}t}+x_{\mathrm{yaw}}(0)(\frac{h_{\mathrm{yr}}}{h_{\mathrm{yy}}-h_{\mathrm{rr}}})(e^{h_{\mathrm{yy}}t}-e^{h_{\mathrm{rr}}t})\\ x_{\text{pitch}}(0)e^{h_{\mathrm{pp}}t}+x_{\mathrm{yaw}}(0)(\frac{h_{\mathrm{yp}}}{h_{\mathrm{yy}}-h_{\mathrm{pp}}})(e^{h_{\mathrm{yy}}t}-e^{h_{\mathrm{pp}}t})\\ x_{\mathrm{yaw}}(0)e^{h_{\mathrm{yy}}t} \end{array})$$

**Figure 3:**
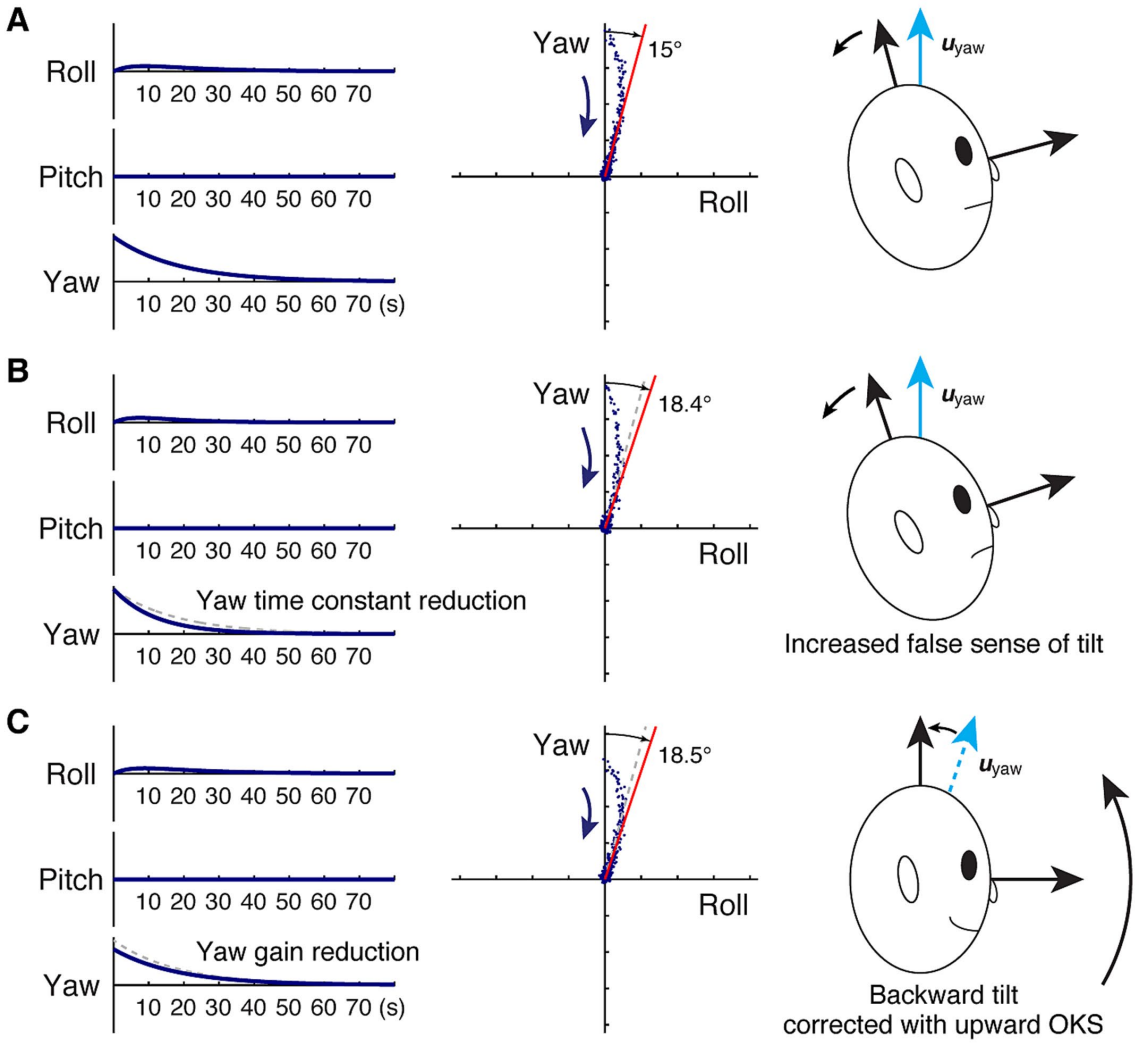
Model-based characterization of backward gravitational pull and changes yielded from velocity storage attenuation. **(A)** Before velocity storage attenuation. **(B)** After velocity storage attenuation, there is a reduction in the yaw time constant. **(C)** After velocity storage attenuation, there is a reduction in the yaw gain. Left panel: idealized roll, pitch, and yaw slow phase velocities subsequent to yaw OKS (zero input response) in an upright position, plotted against time in seconds. The dashed gray traces in **(B,C)** indicate the original response in **(A)**. Center panel: the same response plotted in the pitch-yaw plane. As in Figure 1, each dot represents slow-phase eye velocity sampled at a rate of four per second, but with noise artificially injected to facilitate visualization of spatio-temporal progression in the direction indicated by the arrow. The red line indicates the asymptote, while in **(B,C)**, the dashed gray line indicates that in **(A)**. Right panel: illustration of balance loss caused by the attempt to align the subjective estimate of the up direction with gravity (Figure 2) and a proposed remedy.

We will focus on an idealized OKAN subsequent to yaw OKS (i.e., *x_roll_*(0) = *x_pitch_*(0) = 0), but as with a normal condition, under the stipulation that the yaw time constant is larger than the roll or pitch time constant, the first terms of the roll and pitch components become irrelevant over time as long as *x_yaw_*(0) ≠ 0. The second terms of the roll and pitch components are initially zero because $e^{h_{\mathrm{rr}}t}=e^{h_{\mathrm{pp}}t}=e^{h_{\mathrm{yy}}t}=1$ for *t* = 0. These terms rise until peaking at $t=\frac{\ln(h_{\mathrm{rr}}/h_{\mathrm{yy}})}{h_{\mathrm{yy}}-h_{\mathrm{rr}}}$ and $t=\frac{\ln(h_{\mathrm{pp}}/h_{\mathrm{yy}})}{h_{\mathrm{yy}}-h_{\mathrm{pp}}}$, respectively, and then decline. An example of these dynamics is illustrated in Figure 3A for the roll component associated with backward gravitational pull. Over time, the contribution of the roll or pitch component relative to the yaw component stabilizes. Specifically, given *x_yaw_*(0) ≠ 0, the ratios defined by *x_roll_*(*t*) and *x_pitch_*(*t*) divided by *x_yaw_*(*t*), respectively, approach $\frac{h_{\mathrm{yr}}}{h_{\mathrm{yy}}-h_{\mathrm{rr}}}$ and $\frac{h_{\mathrm{yp}}}{h_{\mathrm{yy}}-h_{\mathrm{pp}}}$ as *t* → ∞. The arctangent of these ratios represents the angle of deviation of the asymptote from the yaw axis in the roll-yaw and pitch-yaw planes, respectively. It is also evident that ***u****_yaw_* represents the asymptote of ***x***.

Using the model, we manipulated the contribution of velocity storage to the central vestibular pathways by changing the time constant or the output weight of the yaw component. The outcomes were examined with changes in the yaw-direction cosine and the zero-input response.

## Results

### Yaw time constant reduction

In the absence of a gravitational pull sensation, i.e., h_yr_ = h_yp_ = 0, as in **H**_**0**_, a change in the yaw time constant does not change the yaw eigenvector, ***u****_yaw_*. On the other hand, with the yaw time constant larger than that of roll or pitch, reducing the yaw time constant to a degree while having other elements of **H**_**pull**_ fixed reduces the difference between h_yy_ and h_rr_ or h_pp._ According to Equation 1, a reduced difference between h_yy_ and h_rr_ or h_pp_ decreases the yaw-direction cosine of ***u****_yaw_*. Therefore, reducing the yaw time constant was found to increase the angle *γ* of misalignment between the head vertical and its velocity storage representation. In terms of zero-input response, reducing the yaw time constant was found to amplify the late contributions of *x_roll_*(*t*) and *x_pitch_*(*t*) relative to *x_yaw_*(*t*), whereby the deviation of the decay trajectory from the yaw axis is increased.

To illustrate the phenomenon, consider the case in which the patient’s subjective estimate of the head vertical is pitched forward by 15° relative to the true head vertical, resulting in a backward pull sensation (Figure 3A). Suppose we start with roll, pitch, and yaw time constants of 5 s, 5 s, and 18 s, respectively, following the observation that the roll and pitch time constants are usually several-fold shorter than the yaw time constant and closer to those of primary afferents when gravity is aligned with the yaw axis (16, 18, 46–48). Then, the system matrix **H**_**pull**_ is given by diagonal elements derived from these time constants, and h_yr_ calculated by solving Equation 1 for *γ* = 15° as:


$$(\begin{array}{ccc} -0.2 & 0 & 0.0378\\ 0 & -0.2 & 0\\ 0 & 0 & -0.0556 \end{array}).$$

The zero-input response with *x_roll_*(0) = *x_pitch_*(0) = 0, i.e., an idealized OKAN response to yaw OKS, has a cross-coupled roll component that initially rises and then declines in addition to the exponentially decaying yaw component. The response follows a curved trajectory in the roll-yaw plane, approaching an asymptote that deviates from the head vertical by 15° as designed.

If we change only h_yy_ to reflect a yaw time constant reduction to, say, 12 s (39–42), **H**_**pull**_ is now:


$$(\begin{array}{ccc} -0.2 & 0 & 0.0378\\ 0 & -0.2 & 0\\ 0 & 0 & -0.0833 \end{array}),$$

resulting in ***u****_yaw_* to deviate from the head vertical by 18.4° per Equation 1, representing an increase in the false sensation of tilt by 24% (Figure 3B). In terms of zero-input response, the faster decline in the yaw component also results in a slightly faster peaking time in the roll component, but the decay profile in the roll-yaw plane becomes stabilized with a larger contribution from the roll component than before the yaw time constant reduction.

Conveniently, however, such an unwanted side effect anticipated with this exploration, when a pull sensation is present, may be circumvented with a separate application of an OKS treatment. For example, for a backward pull sensation, it may be remedied with upward OKS as previously successfully demonstrated in patients with MdDS, presumably by way of nudging the patient’s subjective estimate of the head vertical upward toward the true head vertical (Figure 3C, Left) (35). In the model representation, this remedy is equivalent to reducing the h_yr_ of **H**_**pull**_.

### Yaw gain reduction

Reducing the weight of the yaw component of the velocity storage output is equivalent to the simple coordinate transformation that results in contraction in the yaw dimension or premultiplication of a vector in the original coordinates by:


$$(\begin{array}{ccc} 1 & 0 & 0\\ 0 & 1 & 0\\ 0 & 0 & k \end{array}),\text{where }k\text{ represents the gain}.$$

The system matrix **H**_**pull**_ or its eigenvectors does not change, but the estimate of the head vertical becomes:


$$v_{\mathrm{yaw}}=(\begin{array}{ccc} 1 & 0 & 0\\ 0 & 1 & 0\\ 0 & 0 & k \end{array})u_{\mathrm{yaw}}=(\begin{array}{c} \frac{h_{\mathrm{yr}}}{h_{\mathrm{yy}}-h_{\mathrm{rr}}}\\ \frac{h_{\mathrm{yp}}}{h_{\mathrm{yy}}-h_{\mathrm{pp}}}\\ k \end{array}).$$

Consequently, the angle of misalignment *γ* and the profile of the zero-input response are found to change, for Equations 1, 2, are modified as:


$$\cos(\gamma )=\frac{k}{\sqrt{{\left(\frac{h_{\mathrm{yr}}}{h_{\mathrm{yy}}-h_{\mathrm{rr}}}\right)}^{2}+{\left(\frac{h_{\mathrm{yp}}}{h_{\mathrm{yy}}-h_{\mathrm{pp}}}\right)}^{2}+k^{2}}}$$

and,


$$x(t)=(\begin{array}{c} x_{\text{roll}}(0)e^{h_{\mathrm{rr}}t}+x_{\mathrm{yaw}}(0)(\frac{h_{\mathrm{yr}}}{h_{\mathrm{yy}}-h_{\mathrm{rr}}})(e^{h_{\mathrm{yy}}t}-e^{h_{\mathrm{rr}}t})\\ x_{\text{pitch}}(0)e^{h_{\mathrm{pp}}t}+x_{\mathrm{yaw}}(0)(\frac{h_{\mathrm{yp}}}{h_{\mathrm{yy}}-h_{\mathrm{pp}}})(e^{h_{\mathrm{yy}}t}-e^{h_{\mathrm{pp}}t})\\ kx_{\mathrm{yaw}}(0)e^{h_{\mathrm{yy}}t} \end{array}),$$

with a result of exaggerated *γ* with lower *k*. Note that in the initial absence of a gravitational pull sensation, no new such sensation will be induced by a change in *k*.

Now we again turn to the case of a 15° backward tilt (Figure 3A) with **H**_**pull**_ given by:


$$(\begin{array}{ccc} -0.2 & 0 & 0.0378\\ 0 & -0.2 & 0\\ 0 & 0 & -0.0556 \end{array}).$$

To illustrate the exaggeration of gravitational pull, we reduce the yaw gain by 20%, or set *k* to 0.8 (16). The result is the head vertical being subjectively misestimated by 18.5° and an increased false sensation of backward pull by 25% (Figure 3C). Once again, such an unwanted side effect may be circumvented with OKS to separately correct for a pull sensation, if present (35).

## Discussion

Following the postulate that velocity storage is involved in the pathophysiology of MdDS (13–16, 33–38), we explored two plausible scenarios by which its contribution in the central vestibular pathways may be reduced using a model-based approach, namely, via reduction of the yaw time constant and via reduction of the yaw output gain. When applied to the gravitational pull phenomenon, expressed with yaw-to-roll or yaw-to-pitch coupling in the system matrix of the velocity storage integrator, both scenarios lead to an increase in the misalignment between the head vertical and its velocity storage representation, suggesting symptom *worsening*. Thus, while attenuation of velocity storage may improve symptoms of MdDS in some patients (16), the present model-based exploration points to a possible weakness in this treatment approach as well as an explanation as to why the previous implementation of the approach did not yield a more widely applicable benefit.

We illustrated the effects of velocity storage time constant and gain reduction with their values changing within physiologically plausible ranges reported for human subjects undergoing various vestibular habituation protocols (16, 39–42). On the other hand, we chose an arbitrary angle to illustrate changes in the extent of tilt perception. Such data for patients with MdDS are not available, if it is possible to gather at all. While subjective visual vertical or horizontal may be documented in the roll plane by having a subject adjust an illuminated bar in darkness (49–52), a similar measure is not obtainable currently for the pitch plane. It is also doubtful that a haptic measure of subjective verticality based on an in-hand manipulation of a physical bar or plate will prove to be useful in evaluating a gravitational pull sensation in patients with MdDS, due to the known presence of a bias offset in the measure (51–53). Nevertheless, since the accuracy of subjective visual vertical or horizontal in the roll plane is within 2° in most normal individuals (49–51), just a few degrees of tilt in the subjective estimate of the head vertical may be experienced by patients as gravitational pull. Contrastingly, deviations of 15° or more may be reported by patients with acute unilateral vestibular neurectomy for subjective visual vertical or horizontal in the roll plane (54, 55). While likely overrepresenting the experience of patients with MdDS, we used 15° for illustrative purposes, but the model predicts that a tilt as small as 3–5° still results in a 25% increase with a similar reduction in the velocity storage time constant or gain.

Interindividual differences in the velocity storage time constant and gain are known to be large, and what determines the natural values for a given individual is not known (16, 56–58). Susceptibility to MdDS was previously thought to depend on the strength of velocity storage based on experiments in macaque monkeys that indicated higher susceptibility to three-dimensional spatial maladaptation of the VOR in those with longer velocity storage time constants (33, 59). In contrast, we recently reported that the velocity storage parameters were comparably distributed between patients with MdDS and normal individuals (16). Despite the apparent contradiction, a possibility remains that patients had habituated themselves due to their internally generated self-motion sensation and, in the process, lost the ability to readapt to stable ground and perhaps worsened their symptoms, although means to test for such a speculation are lacking.

Bear in mind that our current exploration provided just an initial estimate of how attenuation of the velocity storage contribution may change the alignment between the head vertical and its velocity storage representation. Previous studies based on data from animals have indicated that a change in the relationship between the head yaw axis and gravity changes roll, pitch, and yaw time constants as well as cross-coupling terms (28, 31, 43). Determining these parameters from human patients is a major challenge, but technological advancements, such as those that allow easy, accurate, and reliable three-dimensional eye movement recording and compelling visual stimuli in a compact head-mounted device, may facilitate such endeavors (60, 61). Furthermore, in our current exploration, attenuation of the velocity storage contribution via reduction of yaw gain was controlled at the output level, although in previous expressions of the velocity storage model, gain adjustments were conceptualized at the level of sensory input (16–18, 31, 35, 43, 62).

Support or revision of the model results may be facilitated by the examination of patient experience regarding gravitational pull after undergoing a velocity storage attenuation protocol. The inferred weakness of a treatment approach for MdDS based on velocity storage attenuation may be remedied with the existing treatment with OKS that specifically targets gravitational pull in MdDS (35). The efficacy of treatment that combines these approaches remains to be tested.

## Data Availability

The original contributions presented in the study are included in the article, further inquiries can be directed to the corresponding author.
